# JunD, not c-Jun, is the AP-1 transcription factor required for Ras-induced lung cancer

**DOI:** 10.1172/jci.insight.124985

**Published:** 2021-07-08

**Authors:** E. Josue Ruiz, Linxiang Lan, Markus Elmar Diefenbacher, Eva Madi Riising, Clive Da Costa, Atanu Chakraborty, Joerg D. Hoeck, Bradley Spencer-Dene, Gavin Kelly, Jean-Pierre David, Emma Nye, Julian Downward, Axel Behrens

**Affiliations:** 1 Adult Stem Cell Laboratory,; 2 Experimental Histopathology, and; 3 Bioinformatics and Biostatistics, The Francis Crick Institute, London, United Kingdom.; 4 Institute of Osteology and Biomechanics, University Medical Center, Hamburg-Eppendorf, Hamburg, Germany.; 5 Oncogene Biology Laboratory, The Francis Crick Institute, London, United Kingdom.; 6 Cancer Stem Cell Laboratory, Institute of Cancer Research, London, United Kingdom.; 7 Imperial College, Division of Cancer, Department of Surgery and Cancer, London, United Kingdom.; 8 Convergence Science Centre, Imperial College, London, United Kingdom.

**Keywords:** Cell Biology, Oncology, Lung cancer

## Abstract

The AP-1 transcription factor c-Jun is required for Ras-driven tumorigenesis in many tissues and is considered as a classical proto-oncogene. To determine the requirement for c-Jun in a mouse model of K-Ras^G12D^–induced lung adenocarcinoma, we inducibly deleted c-Jun in the adult lung. Surprisingly, we found that inactivation of c-Jun, or mutation of its JNK phosphorylation sites, actually increased lung tumor burden. Mechanistically, we found that protein levels of the Jun family member JunD were increased in the absence of c-Jun. In c-Jun–deficient cells, JunD phosphorylation was increased, and expression of a dominant-active JNKK2-JNK1 transgene further increased lung tumor formation. Strikingly, deletion of JunD completely abolished Ras-driven lung tumorigenesis. This work identifies JunD, not c-Jun, as the crucial substrate of JNK signaling and oncogene required for Ras-induced lung cancer.

## Introduction

Abnormal activation of the Ras signaling pathway is commonly found in human tumors. Oncogenic mutations in Ras family proteins, such as K-Ras^G12D^, constitutively activate growth factor signaling pathways and drive uncontrolled cell growth, proliferation, and invasiveness (1–3). Mutations in K-Ras occur in 33% of human lung adenocarcinomas (LADCs) (4), and mutation of K-Ras in the mouse lung is sufficient to induce LADC formation (5). However, since direct targeting of oncogenic Ras has proved extremely challenging, a detailed understanding of Ras downstream pathways is critical to enable targeted therapy for Ras-driven tumors. This need is especially pressing for lung cancer, a tumor type that often responds poorly to current treatments (6).

Among its many outputs, oncogenic Ras signaling stimulates the AP-1 transcriptional activator family, which in turn controls a vast suite of genes involved in proliferation, migration, and apoptosis (7–9). AP-1 family transcription factors are dimeric complexes, composed of various Fos and Jun proteins, which bind a common consensus site known as the TPA response element. The 3 Jun proteins — c-Jun, JunD, and JunB — can either heterodimerize or homodimerize to form an AP-1 complex, and each shows subtle but important differences in regulation and output. JunD- and c-Jun–containing transcription factors are thought to control different, though overlapping, sets of AP-1 target genes (10). In certain contexts, Jun proteins appear to have opposite functions: for example, in immortalized mouse embryonic fibroblasts (MEFs), c-Jun promotes cell proliferation but JunD inhibits it (11, 12). However, each Jun protein can also influence cell behavior in very different ways depending on context — for example, by promoting apoptosis in response to cellular stress or proliferation in response to growth factors (7–9).

Since its discovery as the cellular counterpart of a viral oncoprotein, c-Jun has had a well-established role in tumorigenesis. *c-Jun^–/–^* MEFs display severe proliferation defects and deficiency in cell cycle reentry after serum withdrawal (9, 11, 13). As well as its effects on proliferation through transcriptional upregulation of cell cycle genes such as *cyclinD1* and *cdc2* (9, 14–16), studies in cultured cells demonstrated that c-Jun is required for Ras-mediated oncogenic transformation (17, 18). Conversely, whereas c-Jun cooperates with oncogenic Ras, JunD partially suppresses transformation by activated Ras (19). c-Jun family proteins are activated by phosphorylation mediated by the Jun N-terminal kinases (JNKs) (20). JNK phosphorylates c-Jun on 4 sites — serine 63 (Ser63), Ser73, Ser91, and Ser93 — and JunD on Ser90, Ser100, and Ser117 (21). A mutant allele of c-Jun, in which the JNK phosphoacceptor Ser63 and Ser73 are changed to alanines (Jun^AA^), reduced both oncogenic transformation caused by constitutive activation of the Ras pathway in immortalized fibroblasts and skin tumor development (22), indicating a protumorigenic role of c-Jun N-terminal phosphorylation in Ras-induced transformation. However, the contribution of JunD phosphorylation in the context of oncogenic Ras has not been investigated.

The importance of AP-1 transcription as an output of Ras signaling suggested that c-Jun could be crucial for other tumor types, particularly those driven by oncogenic Ras. Indeed, recent work from our laboratory found that overexpression of the AP-1 coactivator RACO-1 cooperates with oncogenic Ras to increase tumor burden in a mouse model of lung cancer (23, 24). However, the role of c-Jun in lung tumorigenesis was unclear.

In this study, we investigated the importance of different AP-1 components in lung tumorigenesis, using lung-specific deletion of c-Jun or JunD combined with the inducible K-Ras–driven model of LADC (5, 25). Unexpectedly, we found that c-Jun acts as a tumor suppressor in the lung, while JunD is crucial for Ras-driven lung tumor formation. Importantly, JNK signaling appears to be critical in these tumors, as shown by the increased tumor burden observed in a transgenic model of JNK activation and the heightened sensitivity of c-Jun–null lung tumor cells to JNK inhibitors. These data suggest that, in complete contrast to previous findings in other tumor types, c-Jun has tumor-suppressive function in Ras-induced lung tumorigenesis, but paradoxically, JNK should be considered as a therapeutic target in LADC.

## Results

### JUN is frequently lost in human LADC patients.

**Figure 1:**
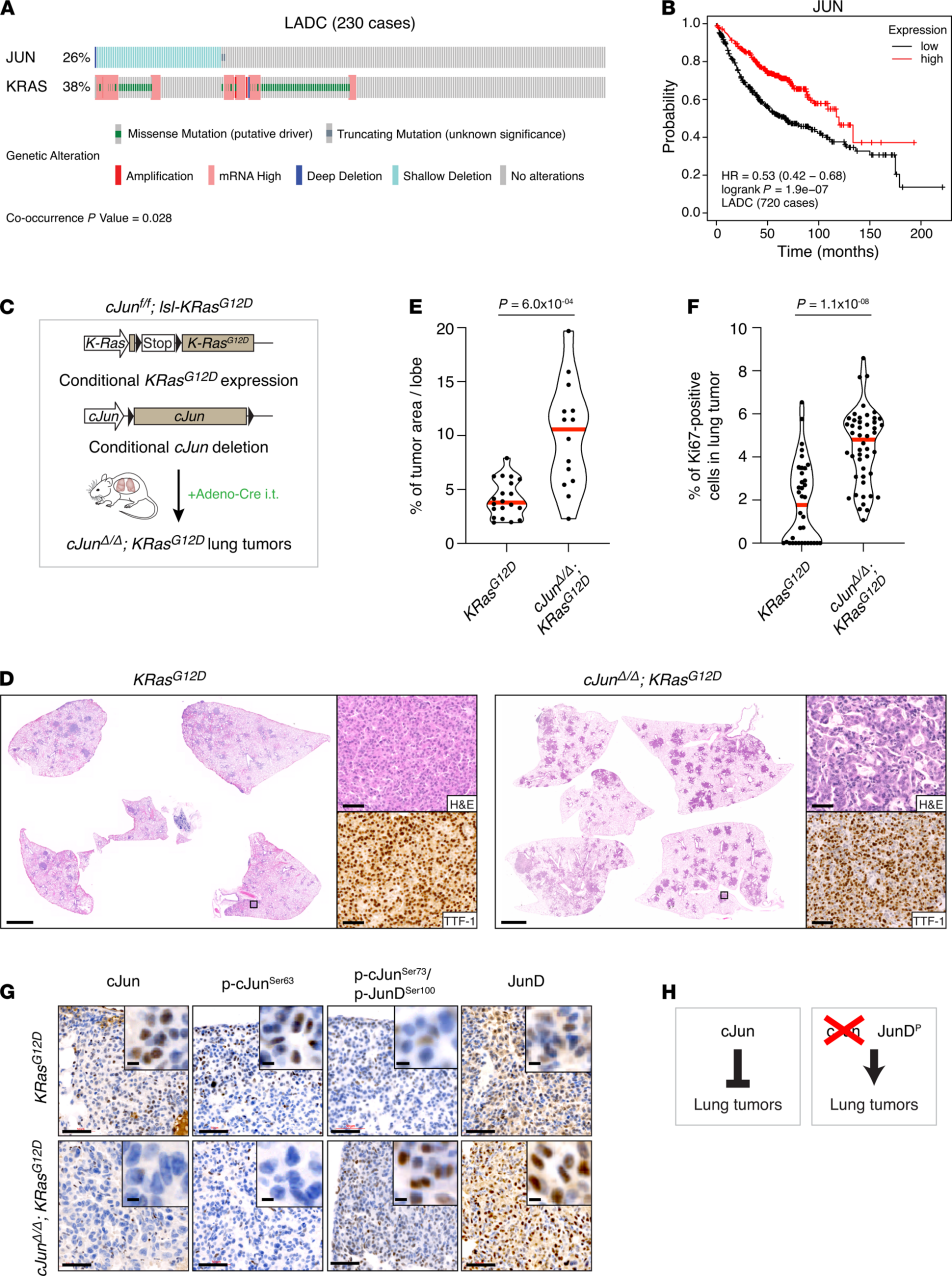
c-Jun is tumor suppressive in the KRasG12D lung adenocarcinoma model. (**A**) Genetic alterations in *JUN* and *KRAS* genes in human LADC. Each column represents a tumor sample (*n* = 230). Data from TCGA were analyzed using cBioportal software. (**B**) Kaplan-Meier plot showing the association between *JUN* expression and patient survival. Analysis performed using KM plotter lung cancer database (cut-off median). (**C**) Schematic representation of *c-Jun^fl/fl^*; *lsl-KRas^G12D^* mouse model. Conditional c-Jun deletion and K-Ras^G12D^ expression in the lung was induced by intratracheal intubation (i.t.) with adenovirus carrying Cre recombinase (Adeno-Cre). LoxP (locus of recombination) sites are indicated by black triangles. LSL, stop cassette flanked by 2 loxP sites. (**D**) H&E and TTF-1 antibody stains of lung sections from mice of the indicated genotypes, 12 weeks after intubation. Scale bars: 2 mm (whole sections), 50 μm (magnified tumor areas). (**E**) Quantification of the tumor burden in whole lungs isolated from *K-Ras^G12D^* (4 mice/20 lobes) and *c-Jun^Δ/Δ^*; *K-Ras^G12D^* (3 mice/14 lobes) mice. Dots, individual lobes; red horizontal line, median. *P* values calculated using unpaired *t* test with Welch’s correction. (**F**) Violin plots quantifying the percentage of Ki67^+^ cells in lung tumors from mice of the indicated genotypes. *K-Ras^G12D^* (4 mice/36 tumors) and *c-Jun^Δ/Δ^*; *K-Ras^G12D^* (3 mice/48 tumors). Each data point represents one tumor. Red horizontal line, median. *P* values calculated using unpaired *t* test with Welch’s correction. (**G**) c-Jun, phospho–c-JunSer63, phospho–c-JunSer73/phospho-JunDSer100, and JunD antibody stains of lung tumors from mice of the indicated genotypes. Scale bars: 50 μm, 5 μm (inset). (**H**) Schematic summary: c-Jun is tumor-suppressor (left). In the absence of c-Jun, K-Ras–dependent tumors increase, possibly through activation of JunD (right). JunD^P^, phospho-JunD.

To evaluate a potential role for genetic disruption of *JUN* in human LADC, we analyzed The Cancer Genome Atlas (TCGA) data from 230 LADC cases. Interestingly, up to 26% of human LADC cases showed loss-of-function genetic alterations (Figure 1A). Moreover, *JUN* and *KRAS* genetic alterations occurred together in a subset of human LADC patients (*P* = 0.028) (Figure 1A). We also examined the correlation between *JUN* expression level and LADC patient survival. Interestingly, lower expression of *JUN* was associated with a significantly shorter survival time (*P* = 1.9 *×* 10^–7^) (Figure 1B). Altogether, these data suggest that *JUN* is often downregulated in human LADC and occurs together with *KRAS* genetic alterations. Therefore, we investigated the functional role of *JUN* in lung cancer.

### c-Jun is tumor suppressive in the K-Ras^G12D^ LADC model.

To determine the role of c-Jun in Ras-driven lung tumorigenesis, we generated mice harboring *c-Jun^fl/fl^* and *lsl-K-Ras^G12D^* alleles and induced c-Jun deletion and K-Ras^G12D^ expression in the lung by intratracheal delivery of adenoviral Cre recombinase (Figure 1C) (25). When analyzed after 12 weeks, both *c-Jun^fl/fl^*; *lsl-K-Ras^G12D^* mice and *lsl-K-Ras^G12D^* controls had developed multiple lung tumors that stained positive for thyroid transcription factor 1 (TTF-1), identifying them as adenocarcinomas (Figure 1D and Supplemental Figure 1A; supplemental material available online with this article; https://doi.org/10.1172/jci.insight.124985DS1). Surprisingly, in c-Jun–deleted lungs, tumor burden was significantly increased and tumors were more proliferative as measured by Ki67 staining (Figure 1, D–F, and Supplemental Figure 1B). The average tumor grade was also increased, with *c-Jun^fl/fl^*; *lsl-K-Ras^G12D^* animals exhibiting grade 4 LADCs, which were HMGA2^+^/TTF-1^–^ and not observed in controls (Supplemental Figure 1, C and D), and some additionally showing lepidic predominant adenocarcinoma (formerly BAC) lesions (Supplemental Figure 1E). To confirm that c-Jun was not active in the observed tumors, we performed IHC staining for c-Jun activating phosphorylation at Ser63 and Ser73 (Figure 1G). Consistent with c-Jun deletion, c-Jun and phospho-Ser63 staining was undetectable in lung tumors from *c-Jun^fl/fl^*; *lsl-K-Ras^G12D^* animals. However, the antibody detecting c-Jun phospho-Ser73, which also recognizes JunD phospho-Ser100, showed increased staining in these tumors. c-Jun–null tumors showed increased staining for JunD (Figure 1G). Thus, in complete contrast to the previously described roles of c-Jun as oncogene and JunD as tumor suppressor (8), Ras-driven lung tumors increase in the absence of c-Jun and show increased JunD phosphorylation and protein expression (Figure 1H).

### JunD phosphorylation is increased in c-Jun–null cells.

**Figure 2:**
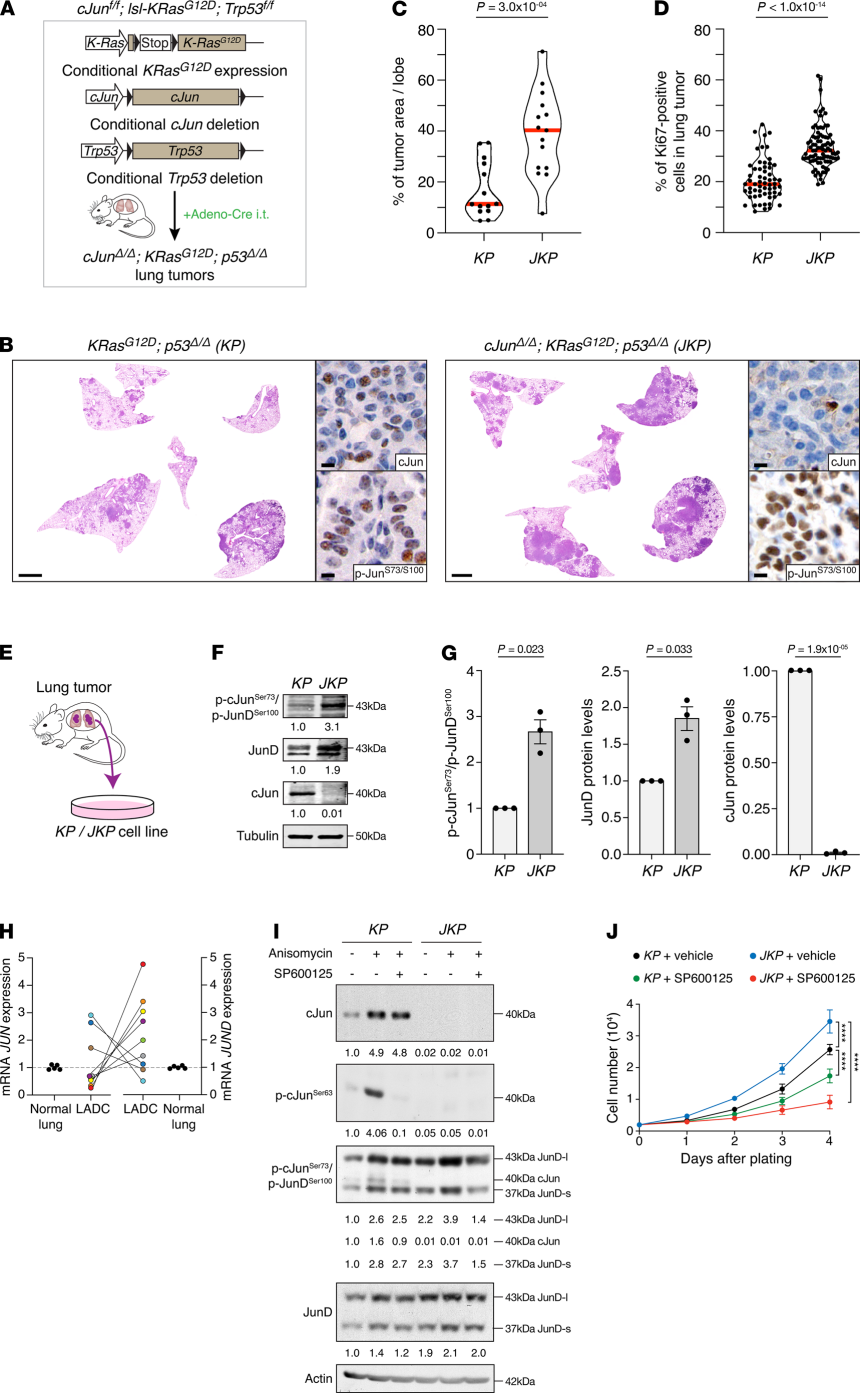
JunD phosphorylation is increased in c-Jun–null cells, which are more sensitive to JNK inhibition. (**A**) Schematic representation of *c-Jun^fl/fl^*; *lsl-KRas^G12D^*; *p53^fl/fl^* mouse model. Conditional c-Jun and p53 deletions and K-Ras^G12D^ expression in the lung was induced by intratracheal intubation (i.t.) with adenovirus carrying Cre recombinase (Adeno-Cre). (**B**) H&E, c-Jun, and phospho–c-JunSer73/phospho-JunDSer100 stains of lung tumors from mice of the indicated genotypes, 10 weeks after intubation. Scale bars: 2 mm (whole sections), 5 μm (inset). (**C**) Quantification of the tumor burden in whole lungs isolated from *KP* (3 mice/14 lobes) and *JKP* (3 mice/15 lobes) mice. Dots, individual lobes; red horizontal line, median. *P* values calculated using unpaired *t* test with Welch’s correction. (**D**) Violin plots quantifying the percentage of Ki67^+^ cells in lung tumors from mice of the indicated genotypes. *KP* (3 mice/60 tumors) and *JKP* (3 mice/85 tumors). Dots, individual lobes; red horizontal line, median. *P* values calculated using unpaired *t* test with Welch’s correction. (**E**) Lung tumor cells from *KP* and *JKP* mice were isolated and cultured in vitro. (**F** and **G**) Immunoblot analysis (**F**) and quantification (**G**) of *KP* and *JKP* cells probed for phospho–c-JunSer73/phospho-JunDSer100, JunD, and c-Jun. The results are expressed as mean ± SEM (*n* = 3 per group). Unpaired *t* test with Welch’s correction. (**H**) Relative mRNA expression of *JUN* and *JUND* in normal lung tissue (*n* = 5) and LADC (*n* = 9) patient samples measured by RT-PCR. Comparison of *JUN* and *JUND* mRNA expression between matched LADC patient samples. (**I**) Immunoblot analysis of *KP* and *JKP* cells with and without anisomycin and JNK inhibitor (SP600125) treatments. (**J**) *KP* and *JKP* cells were cultured with and without JNK inhibitor (SP600125), and proliferation was measured by counting the number of cells. Graph shows means ± SD; ****P < 0.0001, *P* values calculated using 2-way ANOVA with Tukey’s multiple-comparison test.

To investigate the potential oncogenic changes caused by c-Jun deletion in K-Ras mutant lung cells, we isolated primary tumor cells for biochemical analysis. Despite their vigorous growth in vivo, primary tumor cells could not be established from *c-Jun^fl/fl^*; *lsl-K-Ras^G12D^* mice. The severe proliferation defect of c-Jun–deficient primary fibroblasts is rescued by concomitant *Tp53* (*p53*) inactivation (11). Thus, to enable the establishment of primary tumor cell lines in vitro, we combined *c-Jun^fl/fl^*; *lsl-K-Ras^G12D^* alleles with *p53* deletion (Figure 2A). *KRAS* and *TP53* are the most commonly mutated genes in human LADC (4), recapitulated by the widely used *lsl-K-Ras^G12D^*; *p53^fl/fl^* (*KP*) mouse model. As in the p53 WT model, *c-Jun^fl/fl^*; *lsl-K-Ras^G12D^*; *p53^fl/fl^* (*JKP*) mice developed LADCs negative for c-Jun and positive for phospho-JunD (Ser100) (Figure 2B). Tumor area and cell proliferation were significantly increased in *JKP* compared with *KP* mice (Figure 2, C and D, and Supplemental Figure 2A). After isolating individual lung tumors, *KP* and *JKP* cell lines could be established (Figure 2E). We analyzed c-Jun and JunD isoforms that share JNK phosphoacceptor residues recognized by the phospho-Jun (Ser73/Ser100) antibody. Immunoblotting experiments showed that both JunD protein expression and JunD Ser100 phosphorylation rose as a consequence of c-Jun deletion (Figure 2, F and G). Importantly, expression analysis on primary human LADC biopsy samples showed simultaneous downregulation of *JUN* and upregulation of *JUND* (Figure 2H). Moreover, *KP* and *JKP* cells retained their tumorigenicity in culture, as shown by orthotopic transplant of cultured cells directly to the lungs of Nu/Nu mice by intratracheal delivery or s.c. tumor grafts (Supplemental Figure 2, B–F). As expected, tumors derived from *JKP* cells showed increased levels of JunD protein alongside loss of c-Jun immunostaining (Supplemental Figure 2E), and *JKP* tumor–bearing mice succumbed earlier than *KP* mice (Supplemental Figure 2G). Thus, loss of c-Jun expression was associated with a significantly shorter survival time.

### c-Jun–deficient cells are more sensitive to JNK inhibition.

To examine the effects of JNK on c-Jun and JunD in lung tumors, we treated *KP* and *JKP* cells with anisomycin to activate JNK, and we analyzed phosphorylation of c-Jun at Ser63 and Ser73, and phosphorylation of JunD at the analogous Ser100. To be able to distinguish phosphorylation of c-Jun and JunD separately, we ran large gels and separate bands representing c-Jun, and the long and short isoforms of JunD were clearly visible. As expected, anisomycin treatment increased phosphorylation of c-Jun Ser63, as well as increasing total levels of c-Jun in *KP* cells (Figure 2I). JNK-mediated phosphorylation of JunD at Ser100 was more efficient than phospho–c-Jun Ser73 in *KP* cells (Figure 2I and Supplemental Figure 2H). In *JKP* cells, JunD protein and JunD phosphorylation levels were significantly increased, and anisomycin further stimulated phospho–JunD Ser100. Because the total observed phosphorylation detected by the c-Jun Ser73/JunD Ser100 antibody is exclusively due to JunD in *JKP* cells, this result indicates a greater increase in JunD Ser100 phosphorylation in the absence of c-Jun (Figure 2I). Moreover, the increased phospho–JunD Ser100 in anisomycin-stimulated *JKP* compared with *KP* cells was JNK dependent (Figure 2I and Supplemental Figure 2H). This suggests that, in the absence of c-Jun, JNK stimulation of JunD is increased. To test the dependence of *JKP* cells on JNK, we measured the proliferation of primary *KP* and *JKP* cells in the absence or presence of the JNK inhibitor SP600125. In support of an increased role for JNK in supporting proliferation in the absence of c-Jun, *JKP* cells were more sensitive to JNK inhibition than *KP* cells (Figure 2J). Thus, JNK signaling appears to be critical in c-Jun–null lung tumor cells.

### c-Jun^AA^ increases lung tumor burden, phenocopying c-Jun deletion.

**Figure 3:**
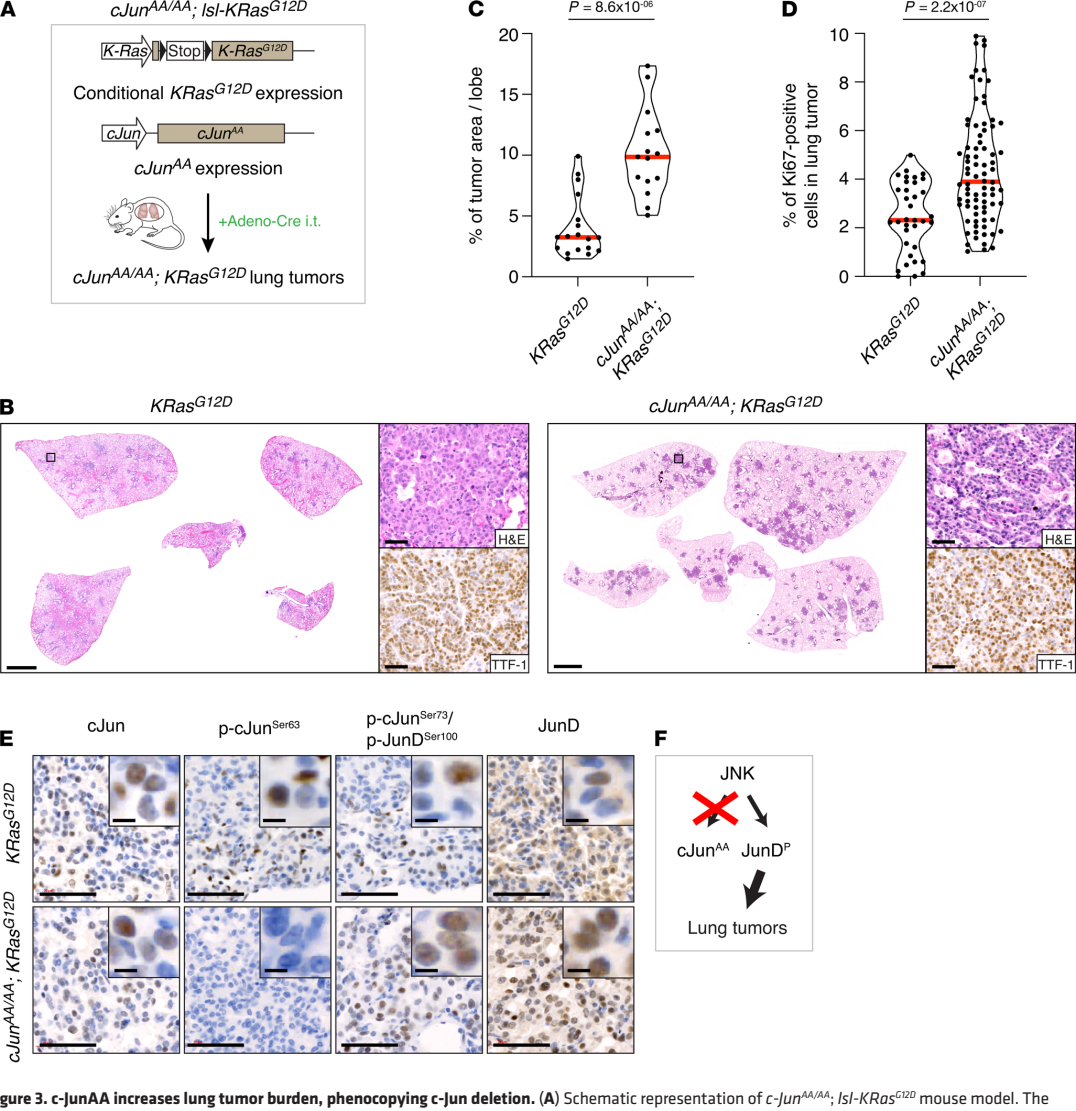
c-JunAA increases lung tumor burden, phenocopying c-Jun deletion. (**A**) Schematic representation of *c-Jun^AA/AA^*; *lsl-KRas^G12D^* mouse model. The c-JunAA allele lacks the JNK phosphorylation sites Ser63 and Ser73. Expression of K-Ras^G12D^ was induced by intratracheal intubation (i.t.) with adenovirus carrying Cre recombinase (Adeno-Cre). LSL, stop cassette flanked by 2 loxP (locus of recombination) sites. (**B**) H&E and TTF-1 antibody stains of lung sections from mice of the indicated genotypes, 12 weeks after intubation. Scale bars: 2 mm (whole sections), 50 μm (magnified areas). (**C**) Quantification of the tumor burden in whole lungs isolated from *K-Ras^G12D^* (4 mice/18 lobes) and *c-Jun^AA/AA^*; *K-Ras^G12D^* (3 mice/15 lobes) mice. Dots, individual lobes; red horizontal line, median. *P* values calculated using unpaired *t* test with Welch’s correction. (**D**) Violin plots quantifying the percentage of Ki67^+^ cells in lung tumors from mice of the indicated genotypes. *K-Ras^G12D^* (3 mice/35 tumors) and *c-Jun^AA/AA^*; *K-Ras^G12D^* (3 mice/80 tumors). Each data point represents 1 tumor. Red horizontal line, median. *P* values calculated using unpaired *t* test with Welch’s correction. (**E**) c-Jun, phospho–c-JunSer63, phospho–c-JunSer73/phospho-JunDSer100, and JunD antibody stains of lung tumors from mice of the indicated genotypes. Scale bars: 50 μm, 5 μm (inset). (**F**) Schematic summary: Blocking JNK phosphorylation of c-Jun increases oncogenic K-Ras–dependent lung tumor burden, similar to the effects of c-Jun deletion, possibly via increased phosphorylation of JunD.

To test the idea that JNK-mediated phosphorylation is important for tumorigenesis, we used the partially inactive c-Jun S63A, S73A N-terminal phosphorylation mutant, termed c-Jun^AA^. This mutant lacks 2 JNK phosphorylation sites and shows impaired, but not abolished, AP-1 transactivation (26). We generated *c-Jun^AA/AA^*; *lsl-K-Ras^G12D^* mice and induced tumorigenesis by administration of adenoviral Cre to the lung, as before (Figure 3A). The presence of mutant c-Jun did not impair tumorigenesis; in fact, lung tumor burden and cell proliferation in tumors were increased, as in the c-Jun deletion model (Figure 3, B–D). Thus, despite the presence of c-Jun protein, as detected by IHC (Figure 3E), impaired JNK phosphorylation of c-Jun was sufficient to increase Ras-driven lung tumorigenesis (Figure 3F). Interestingly, JunD levels were also increased in *c-Jun^AA/AA^*; *lsl-K-Ras^G12D^* mice, similar to the c-Jun deletion model (Figure 3E). As JunAA still binds and induces its target promoters (26), the observed *c-Jun*–null–like (*c-Jun*^ΔL^**–like) phenotype observed in *c-Jun^AA/AA^*; *lsl-K-Ras^G12D^* mice suggests an essential role for JNK signaling.

### JNK activity is protumorigenic in the absence of c-Jun.

**Figure 4:**
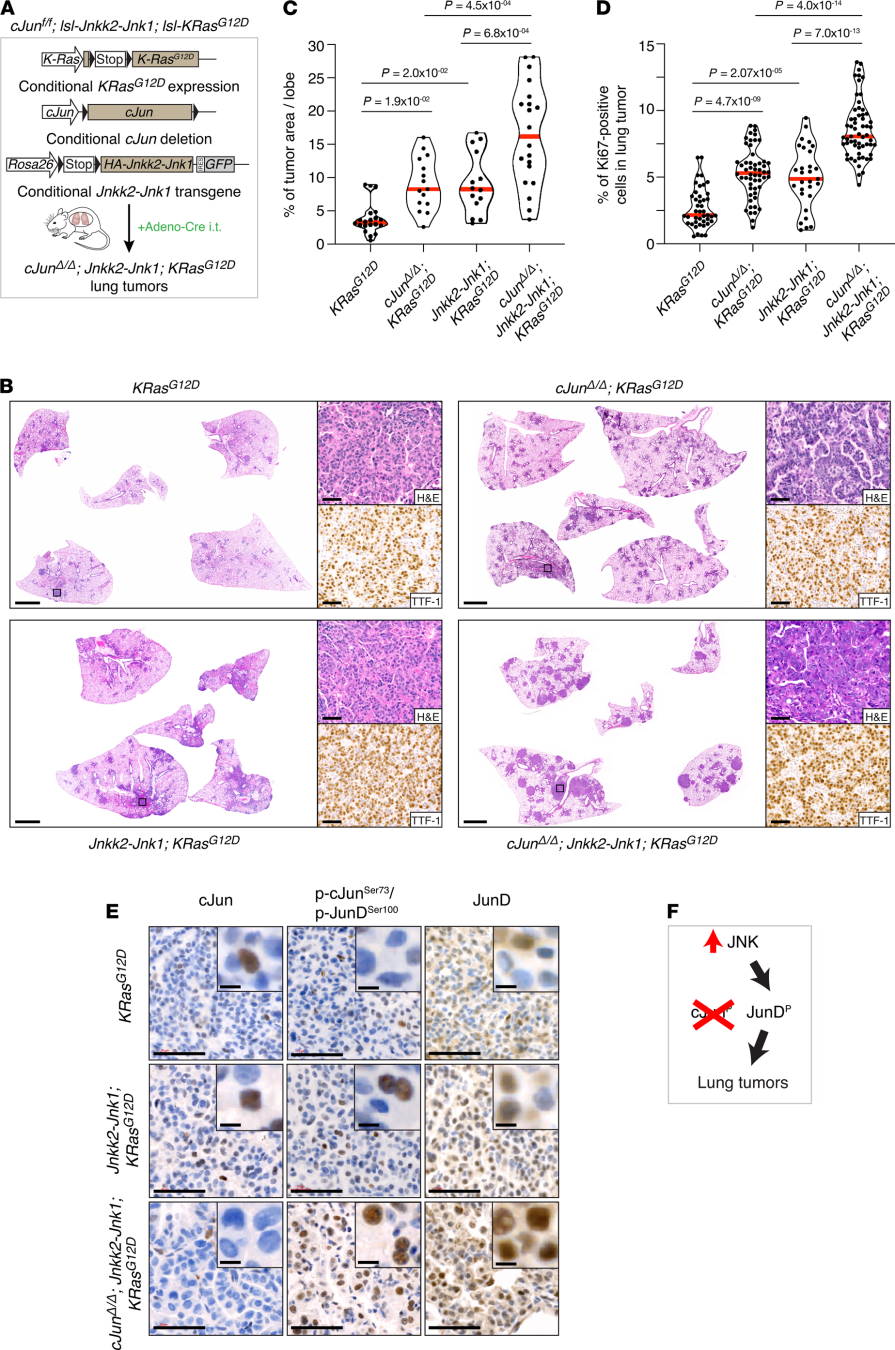
JNK activity is protumorigenic in the absence of c-Jun. (**A**) Schematic representation of *c-Jun^fl/fl^; lsl-Jnkk2-Jnk1; lsl-K-Ras^G12D^* mouse model. Conditional c-Jun deletion and expression of K-Ras^G12D^ and the Jnkk2-Jnk1 transgene (described in Supplemental Figure 3) in the lung was induced by intratracheal intubation (i.t.) with adenovirus carrying Cre recombinase (Adeno-Cre). LSL, stop cassette flanked by 2 loxP (locus of recombination) sites (black triangles); IRES, internal ribosome entry site. (**B**) H&E and TTF-1 antibody stains of lung sections from mice of the indicated genotypes, 12 weeks after intubation. Scale bars: 2 mm (whole sections), 50 μm (magnified areas). (**C**) Quantification of the tumor burden in whole lungs isolated from *K-Ras^G12D^* (4 mice/20 lobes), *c-Jun^Δ/Δ^*; *K-Ras^G12D^* (3 mice/15 lobes)*, Jnkk2-Jnk1*; *K-Ras^G12D^* (3 mice/14 lobes)*,* and *c-Jun^Δ/Δ^*; *Jnkk2-Jnk1; K-Ras^G12D^* (4 mice/20 lobes) mice. Dots, individual lobes; red horizontal line, median. *P* values calculated using 1-way ANOVA with Tukey’s multiple-comparison test. (**D**) Violin plots quantifying the percentage of Ki67^+^ cells in lung tumors from mice of the indicated genotypes. *K-Ras^G12D^* (4 mice/43 tumors), *c-Jun^Δ/Δ^*; *K-Ras^G12D^* (3 mice/54 tumors)*, Jnkk2-Jnk1*; *K-Ras^G12D^* (3 mice/29 tumors)*,* and *c-Jun^Δ/Δ^*; *Jnkk2-Jnk1; K-Ras^G12D^* (4 mice/59 tumors). Each data point represents 1 tumor. Red horizontal line, median. *P* values calculated using 1-way ANOVA with Tukey’s multiple-comparison test. (**E**) c-Jun, phospho–c-JunSer73/phospho-JunDSer100, and JunD antibody stains of lung tumors from mice of the indicated genotypes. Scale bars: 50 μm, 5 μm (inset). (**F**) Schematic summary: JNK overexpression in the absence of c-Jun promotes oncogenic K-Ras–dependent tumorigenesis.

To test JNK activity in vivo, we combined inducible *K-Ras^G12D^* expression and *c-Jun* deletion in the lung with a conditional JNKK2-JNK1 transgene to augment JNK activity (Figure 4A and Supplemental Figure 3, A–D). Expression of the transgene increased K-Ras^G12D^–driven tumor burden and tumor cell proliferation, suggesting that JNK activity is protumorigenic (Figure 4, B–D, and Supplemental Figure 3E). Tumor burden was significantly (*P* = 4.5 × 10^–4^) increased in the lungs of *c-Jun^fl/fl^*; *lsl-Jnkk2-Jnk1*; *lsl-K-Ras^G12D^* mice compared with *c-Jun^fl/fl^*; *lsl-K-Ras^G12D^* mice, suggesting that c-Jun loss and increased JNK activity cooperate to promote tumorigenesis (Figure 4B and Supplemental Figure 3F). Consistent with JunD as a key effector of JNK, JunD levels were increased in correlation with the increased tumor burden in *c-Jun^fl/fl^*; *lsl-Jnkk2-Jnk1*; *lsl-K-Ras^G12D^* lungs, with positive staining for phospho-JunD Ser100 (Figure 4E). Thus, although c-Jun is tumor suppressive in the context of Ras-driven lung tumorigenesis, JNK signaling appears to be oncogenic, and increased JunD phosphorylation by JNK is a potent tumor promoter in the lung (Figure 4F).

### JunD is required for Ras-driven lung tumorigenesis.

To investigate the correlation between *JUND* levels and patient survival, we examined *JUND* expression from a transcriptomic data set of a human LADC cohort. We found that high *JUND* expression is significantly correlated with decreased overall survival for patients with LADC (*P* = 3.3 *×* 10^–4^) (Supplemental Figure 4A).

**Figure 5:**
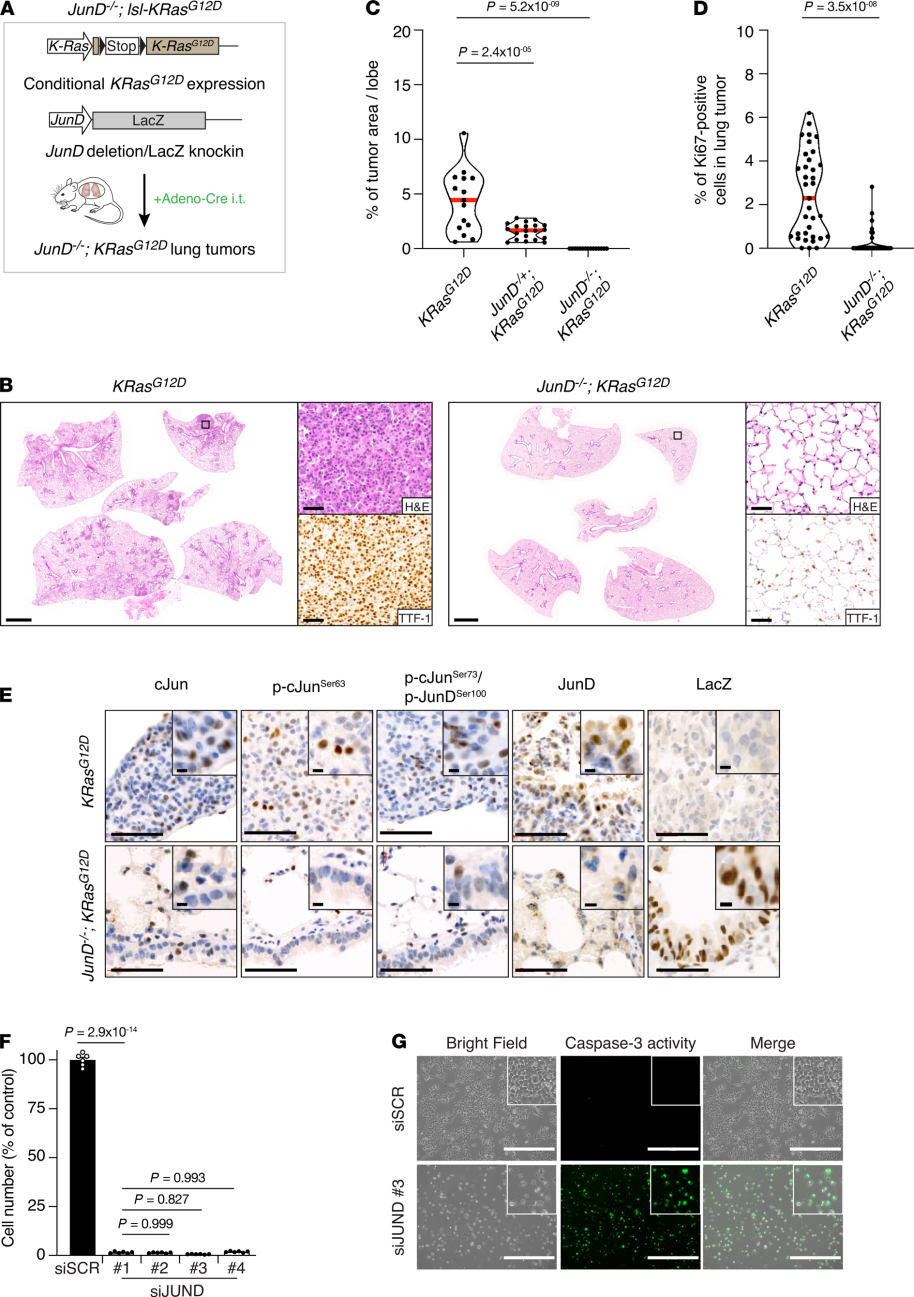
JunD is required for Ras-driven lung tumorigenesis. (**A**) Schematic representation of *JunD^–/–^*; *lsl-KRas^G12D^* mouse model. K-Ras^G12D^ expression was induced by intratracheal intubation (i.t.) with adenovirus carrying Cre recombinase (Adeno-Cre). LSL, stop cassette flanked by 2 loxP (locus of recombination) sites. (**B**) H&E and TTF-1 antibody stains of lung sections from mice of the indicated genotypes, 12 weeks after intubation. Scale bars: 2 mm (whole sections), 50 μm (magnified areas). (**C**) Quantification of the tumor burden in whole lungs isolated from *K-Ras^G12D^* (3 mice/15 lobes), *JunD^–/+^*; *K-Ras^G12D^* (4 mice/20 lobes)*,* and *JunD^–/–^*; *K-Ras^G12D^* (3 mice/15 lobes) mice. Dots, individual lobes; red horizontal line, median. *P* values calculated using 1-way ANOVA with Tukey’s multiple-comparison test. (**D**) Violin plots quantifying the percentage of Ki67^+^ cells in lung tumors from mice of the indicated genotypes. *K-Ras^G12D^* (3 mice/35 tumors), *JunD^–/–^*; *K-Ras^G12D^* (3 mice/67 untransformed lung regions). Each data point represents 1 untransformed area or tumor. Red horizontal line, median. *P* values calculated using unpaired *t* test with Welch’s correction. (**E**) c-Jun, phospho–c-JunSer63, phospho–c-JunSer73/phospho-JunDSer100, JunD, and LacZ antibody stains of lung tumors from mice of the indicated genotypes. Scale bars: 50 μm, 5 μm (inset). (**F**) Graph showing the difference in cell proliferation between control (Scramble) and siJUND-transfected human NCI-H23 LADC cells. Graph indicates mean ± SEM. *P* values calculated using 1-way ANOVA with Tukey’s multiple-comparison test. (**G**) Impaired cell growth in siJUND-transfected NCI-H23 LADC cells is associated with apoptotic cell death based on the DEVD-NucView488 Caspase-3 dye that detects caspase-3 activity. Scale bars: 400 μm.

To directly test the role of JunD in Ras-driven lung tumorigenesis, we combined inducible *K-Ras^G12D^* expression in the lung with JunD germline KO (Figure 5A). Strikingly, *K-Ras^G12D^* was unable to induce lung tumors in *JunD^–/–^* mice, and cell proliferation in lungs was correspondingly reduced (Figure 5, B–D, and Supplemental Figure 4B). Since *JunD^–/–^* animals show postnatal growth defects, we also examined *JunD* heterozygotes, which are indistinguishable in size from WT littermates (27). JunD heterozygous mice expressing *K-Ras^G12D^* in the lung also showed a significantly reduced tumor burden compared with JunD-proficient controls (Figure 5C), suggesting that JunD plays a driving role in K-Ras^G12D^–induced tumorigenesis. We confirmed loss of JunD and expression of the LacZ knockin by IHC (Figure 5E).

As JunD was knocked out throughout the whole mouse, there was the possibility that nontumor cells may have played a role during tumorigenesis. Thus, to confirm a function of JunD in lung cancer cells, JunD protein expression was knocked out via CRISPR/Cas9 in primary lung *KP* cells. JunD-null cells (*KPD*) proliferated slower than parental *KP* controls (Supplemental Figure 4C). When injected s.c. into nude mice, JunD-deficient cells formed graft tumors that were smaller and grew slower than those derived from *KP* cells (Supplemental Figure 4, D and E). Importantly, *JUND* downregulation by siRNA severely reduced cell proliferation in human LADC cell lines (Figure 5F and Supplemental Figure 5, A and B). The loss of cell growth was associated with apoptotic cell death, as detected by caspase-3 activity probes (Figure 5G and Supplemental Figure 5C). Thus, these data suggest a role for JunD in lung tumorigenesis.

We next performed immunoblotting analysis for Jun protein levels and activating phosphorylation at c-Jun Ser63 and c-Jun Ser73/JunD Ser100. Surprisingly, c-Jun protein levels, phospho–c-Jun Ser63, and phospho–c-Jun Ser73 levels were significantly reduced in both mouse and human JunD–deficient cells (Supplemental Figure 4, F and G, and Supplemental Figure 5B). IHC analysis in *JunD^–/–^; lsl-K-Ras^G12D^* lungs confirmed reduction in phospho–c-Jun Ser63 and phospho–c-Jun Ser73 (Figure 5E). Importantly, immunoblotting experiments did not show any alteration in JNK protein and active JNK phospho-JNK^Thr183/Tyr185^ levels in JunD-deficient cells (Supplemental Figure 4, F and G). These results suggest that JNK activity is unaltered in JunD-KO cells.

As lung tumorigenesis appears to be JunD dependant, we performed RNA-sequencing (RNA-Seq) experiments using primary *KP* and *JKP* tumor cells to gain mechanistic insights. Gene set enrichment analysis (GSEA) found that oxidative stress, adipogenesis, and p38 MAPK pathways were upregulated in *JKP* cells (Supplemental Figure 6A), where JunD phosphorylation and protein expression were increased (Figure 2F). These data are in agreement with a previous study that indicated JunD works as an activator of transcription of genes involved in oxidative stress, differentiation, and cell proliferation (28). We further confirmed activation of p38α MAPK in *JKP* cells (Supplemental Figure 6B), which correlated with increased JunD protein levels (Supplemental Figure 6C). Therefore, *JKP* cells were sensitive to p38 MAPK inhibition (Supplemental Figure 6D). By contrast, p38α MAPK activation was reduced in JunD-deficient cells (Supplemental Figure 6C). In addition, analysis of public gene expression databases revealed a positive correlation between the expression of *JUND* and *MAPK14* in human LADC samples (Supplemental Figure 6E). Interestingly, p38α protein and phosphorylation levels are increased and correlate with poor survival in LADC patients (29, 30). Moreover, p38α supports the progression of K-Ras–driven lung tumors (30). Thus, p38α MAPK pathway activation could contribute to the effects of JunD as a lung tumor promoter.

Overall, these results support a tumor-suppressive role for c-Jun in the lung and demonstrate that JunD is required for Ras-driven lung tumorigenesis.

## Discussion

Unexpectedly, we found that c-Jun, a classic proto-oncogene, has a tumor-suppressive effect in a well-established model of LADC. These results are surprising, since c-Jun was shown to cooperate with oncogenic K-Ras in promoting proliferation of cultured fibroblasts, and because of the requirement for c-Jun in skin, liver, and intestinal Ras-dependent tumor models (17, 18, 22, 31, 32).

Instead, our results shown that JunD is essential for lung tumorigenesis. It is tempting to speculate that JNK signaling has tumor-suppressive effects through c-Jun and tumor-promoting effects through JunD and that loss of one arm tips the balance toward the effects of the other arm. To investigate the possibility that increased JunD function underlies the increased lung tumorigenesis in the absence of *c-jun*, we aimed to knock out JunD in *JKP* cells through CRISPR-meditated genome editing. Unfortunately, c-Jun/JunD–double KO cell lines could not be generated, possibly because JunD function is essential in *JKP* cells (data not shown). However, we successfully derived *KP* cells deficient in JunD through CRISPR, and these cells showed a clear growth disadvantage.

Our results show that c-Jun protein and phosphorylation levels are reduced in the absence of JunD. By contrast, JunD protein and phosphorylation levels rise in the absence of c-Jun. JNK signaling was shown to have tumor-promoting and tumor-suppressive functions in tumorigenesis. A previous study found that JNK is essential for Ras-driven lung tumorigenesis, but the relevant JNK substrates were not identified (33). In other systems, such as prostate and breast cancers, JNK has been shown to be tumor suppressive (34, 35), while in the liver, JNK has both pro- and antitumorigenic roles (36). It is possible that the balance between c-Jun and JunD contributes to the variation in JNK signaling outcome in these different contexts. Our data suggest that JNK activity is protumorigenic in absence of c-Jun through JunD. JunD is phosphorylated by ERK, as well as JNK (37), so ERK-driven function may contribute to JunD’s role in K-Ras–driven tumors. Indeed, JunD increases prostate cancer cell migration in an ERK-dependent manner (10). We also do not discard the possibility that c-Jun loss and/or JNK activation additionally promote tumorigenesis by JunD-independent mechanisms. However, our in vivo data indicate that JunD is absolutely required for K-Ras–driven lung tumorigenesis, highlighting JunD as an important protumorigenic JNK target.

## Methods

### Analysis of public data from cancer genomics studies.

Data from TCGA Research Network (TCGA Lung Adenocarcinoma), including mutations and putative copy number alterations, were analyzed using cBioportal software and visualized using the standard Oncoprint output. Patient prognoses was evaluated by Kaplan-Meier survival curves of LADC patients with low or high expression of *JUN* and *JUND*, with data from Kaplan–Meier plotter. Correlation analysis were performed using GEPIA (Gene expression profiling interactive analysis) software.

### Mice.

Nu/Nu mice were derived from the colony established by the Imperial Cancer Research Fund (ICRF nude). *c-Jun^fl/fl^* (38), *lsl-KRas^G12D^* (5), *p53^fl/fl^* (39), *c-Jun^AA^* (26), and *JunD*^Δ*/*Δ^** (27) animals have been described before. Generation of the lsl-Jnkk2-Jnk1 transgenic construct is described in Supplemental Figure 3. Constructs were electroporated into ES cells, stable transfectants were selected, and mice were generated by injection of a positive ES cell clone into C57BL/6 blastocysts according to standard protocols (26).

### Adenoviral infection.

Adeno-CMV-Cre virus (Ad5CMVCre) was purchased from the Gene Transfer Vector Core, University of Iowa (Iowa City, Iowa, USA). Intratracheal intubation was carried out on animals at 8–12 weeks of age using a dose of 2.5 × 10^7^ viral particles per mouse as described (25). Briefly, the adenovirus was prepared in PBS containing 10 μM calcium chloride and magnesium. Mice were anesthetized with isoflurane, and the virus preparation was administered via a catheter inserted into the trachea. Both male and female mice were used. A sample size of at least 3 mice per genotype was used, with no sample exclusion criteria. Mice were culled 12 weeks after intubation for analysis. The effect size was not prespecified. No animals were excluded from the analysis, and randomization was used.

### S.c. graft tumors.

Lung *KP, JKP,* and *KPD* tumor cells were resuspended as single-cell suspensions at 1 × 10^5^ cells/mL in PBS/Matrigel (Corning, 354230). In total, 100 μL (1 × 10^4^ cells total) of this suspension was injected into opposite left (*KP*) and right (*KPD*) flanks of athymic Nu/Nu nude mice. For survival curves, *KP* and *JKP* cells were injected in individual nude mice. Tumor grafts were measured with digital calipers, and tumor volumes were determined with the following formula: (length × width^2^) × (π/6). Tumor volumes are plotted as means ± SEM.

### Orthotopic lung tumors.

Lung *KP* and *JKP* tumor cells were resuspended as single-cell suspensions at 1 × 10^6^ cells/50 μL in PBS and delivered directly to the lungs of 8-week-old Nu/Nu by intratracheal intubation. Lungs were analyzed after 12 weeks after intubation.

### Histological analysis.

Animals were euthanized by cervical dislocation, and the lung and heart were prepared for histopathological analysis as previously described (24). In short, excised lungs were incubated in ice-cold endotoxin-free PBS, then in 10% NBF overnight, and then in 70% industrial methylated spirit until further processing. Paraffin-embedded 4 μm sections were stained with the following antibodies: TTF-1 (ab40880, Abcam), LacZ (AB9361, Abcam), c-Jun (610326, BD Biosciences), phospho–c-Jun Ser63 (9261, NEB), phospho–c-Jun Ser73 (9164, NEB), JunD (sc74, Santa Cruz Biotechnology Inc.), HMGA2 (8179, Cell Signaling Technology [CST]), and Ki67 (M7248, DAKO).

For quantification of tumor burden, the area (μm^2^) of existing individual tumors in each lobe was measured with QuPath software (40) (Measure > Show Annotation Measurements Tool) and represented as a percentage of tumor area per lobe. The analysis was performed uniformity across all lung sections, and the whole lungs were used to derive data. For quantification of cell proliferation, the number of Ki67^+^ cells was automatically counted in individual tumors with QuPath software (Cell Analysis > Positive Cell Detection Tool) and represented as a percentage of total cells in tumor area.

### Isolation of primary lung tumor cells.

Primary murine LADC cells were obtained as previously described (41). Lung lobes were dissected out and immediately placed in 4 mL of medium A (AdMEM/F12, B27, N2, 2% FCS, 1% penicillin/streptomycin, 10 μg/mL insulin, 20 ng/mL EGF, 20 ng/mL FGF, 100 μg/mL Primocin) into GentleMACS C tubes and subjected to automated homogenization using GentleMACS dissociators at Tumor 2 mode, followed by addition of 300 U/mL collagenase IV (Worthington, LS004186) and hyaluronidase 300 U/mL (Worthington, LS002594), as well as incubation for 20 minutes at 37°C with constant shaking. Homogenized lungs were subjected to further dissociation at Tumor 3 mode, followed by centrifugation at 150*g* for 5 minutes at 4°C. The pellet was resuspended in medium A, and cells were plated onto Ultra-low attachment plates and placed in a humidified incubator at 37°C, 5% CO_2_. Ninety-six hours later, the medium was replaced with DMEM (10% FCS, 1% penicillin/streptomycin), and cells were replated on standard tissue culture plates. All cell lines were tested as mycoplasma negative by PCR assay.

### Cell culture.

Primary murine lung cells were maintained at 37°C with 5% CO_2_. We noticed that *JKP* cells maintained in culture for a prolonged period of time (passage > 15) lose their growth advantage over *KP* cells. Therefore, all experiments were performed using cells from passages 3–10. Where indicated, cells were treated with anisomycin (MilliporeSigma, A9789, final concentration 200 ng/mL) for 30 minutes to stimulate JNK-dependent phosphorylation of c-Jun/JunD, and/or with JNK inhibitor (Tocris Bioscience, Bio-Techne; SP600125; final concentration 20 μM) for 30 minutes before harvesting (after anisomycin stimulation, if given).

Human LADC cell lines (NCI-H23, NCI-H441, NCI-H1792, and A549) were provided by the Francis Crick Institute Cell Services and cultured in RPMI-1640 medium supplemented with 10% FBS, 1% penicillin/streptomycin, and 2mM glutamine. All cells were tested Mycoplasma negative and maintained at 37°C with 5% CO_2_.

### Immunoblotting.

For protein analyses of lung tumor cell lines, cells were harvested, washed in PBS, and resuspended in cell lysis buffer (9803, NEB) supplemented with protease inhibitor cocktail (MilliporeSigma). Protein amount was measured by Lowry assay. The following antibodies were used for Western blot analyses: c-Jun (610326, BD Biosciences), phospho–c-Jun Ser63 (9261, New England Biolabs [NEB]), phospho–c-Jun Ser73 (9164, NEB), JunD (sc74, Santa Cruz Biotechnology Inc.), JunD (5000, NEB), phospho–SAPK/JNK Thr183/Tyr185 (4668 and 9251, NEB), SAPK/JNK (9252, NEB), phospho–p38 MAPK Thr180/Tyr182 (9211, NEB), p38 MAPK (9212, NEB), tubulin (ab7291, Abcam), and β-actin (ab49900, Abcam). All primary antibodies were used at 1:1000 dilution. Horseradish peroxidase–conjugated (HRP-conjugated) secondary antibodies were used at 1:5000 dilution, and anti–β-actin HRP antibody was used at 1:30,000 dilution. For Western blot utilizing the LI-COR system, DyLight goat anti–mouse 680 (5470, NEB) and DyLight goat anti–rabbit 800 (5151, NEB) secondary antibodies were used at 1:15,000 dilution. Separate immunoblots were used for each antibody.

### Cell proliferation assay.

Primary murine lung cells were seeded in 96-well plates at a density of 2 × 10^3^ cells per well. Twelve hours later, cells were treated with DMSO as vehicle, JNK inhibitor (Tocris Bioscience, Bio-Techne; SP600125, 20 μM), or p38 MAPK inhibitor (MilliporeSigma, SB203580, 10 μM) throughout the experiment. Cell confluence was measured over several days, using a hemocytometer. Alternatively, cell viability was measured as the intracellular ATP content using the CellTiter-Glo Luminescent Cell Viability Assay (Promega), following the manufacturer’s instructions.

### JUND silencing and imaging apoptosis.

Human LADC cell lines (NCI-H23, NCI-H441, NCI-H1792, and A549) were transfected with specific small interfering RNAs (siRNAs) against the *JUND* gene, using Lipofectamine RNAiMAX and 25 nM of each siRNA according to the manufacturer’s instructions (Dharmacon) (42). Detection of caspase-3 activity in cells was achieved through incubation with the cell-permeable caspase-3 substrate DEVD-NucView488 generated in-house by the Peptide Chemistry Laboratory as described (43). Forty-eight to 72 hours later, caspase-3 enzyme activity was detected using fluorescence microscopy, and cell number was counted using an automated cell counter.

### Crispr/Cas9 deletion of JunD.

Guide RNA sequences targeting murine JunD were designed using the CHOPCHOP design tool (http://chopchop.cbu.uib.no). Two guide RNAs were designed with a spanning sequence of 200 bp. Guide RNA sequences were cloned into px458 and px459 (Addgene plasmids 48138 and 48139, respectively). *KP* cells were transiently cotransfected with both guide RNAs, and 48 hours after transfection, cells were selected with 4 μg/mL puromycin for 72 hours. Single clones were then propagated in medium lacking puromycin and analyzed by immunoblot.

### RNA-Seq/GSEA.

*KP* and *JKP* cells were lysed with 600 μL 1% β-Mercaptoethanol in buffer RLT (QIAGEN). RNA was extracted using the RNeasy Mini Kit (QIAGEN) according to the manufacturer’s instructions. mRNA-Seq libraries were prepared using TruSeq RNA Library Preparation Kit v2 (Illumina) according to the manufacturer’s instructions. Libraries were sequenced using the HiSeq2500 systems (Illumina). Sequenced reads were mapped and quantified to mm10 whole genome and refseq transcript RSEM v1.2.11 (including Bowtie v2.1.0) with parameters --bowtie2-sensitivity-level very_sensitive --estimate-rspd. RNA-Seq data were deposited in the Gene Expression Omnibus repository under accession number GSE87387.

GSEA was performed on the transcription profile of *KP* and *JKP* cells with the software GSEA v4.0.3. The settings in analyses were: number of permutations = 1000, permutation type = gene set. The following gene set databases were used: Hallmarks and Biocarta.

### Human study approval and lung tumor analysis.

Human biological samples were collected, stored, and managed by the Cordoba node belonging to the Biobank of the Andalusian Health Service (Servicio Andaluz de Salud — SAS) and approved by the Ethics and Clinical Research Committee of the University Hospital Reina Sofia. All subjects gave informed consent. Pathologists assessed all samples before use. mRNA extracted from the samples was analyzed by quantitative PCR. The primers were as follows: Actin forward, 5′‑GAAAATCTGGCACCACACCT‑3′, and reverse, 5′‑TAGCACAGCCTGGATAGCAA‑3′; JUN forward, 5′‑CCAAAGGATAGT GCGATGTTT‑3′, and reverse, 5′‑CTGTCCCTCTCCACTGCAAC‑3′; JUND forward, 5′‑CAGCGAGGAGCAGGAGTT‑3′, and reverse, 5′‑GAGCTGGTTCTGCTTGTGTAAAT‑3′.

### Statistics.

To analyze tumor area and tumor cell proliferation, a 2-tailed Welch’s *t* test was used to compare data from 2 different genotypes. This test does not assume equal variances and performs better when sample sizes are unequal. One-way or 2-way ANOVA was used to compare data from 3 or more different groups. Data distribution was not formally analyzed. Estimates of variation (range, SD, or SEM as specified in the figure legends) are given in the figures. A *P* value less than 0.05 was considered significant.

### Study approval.

All animal experiments were approved by the Francis Crick Institute Animal Ethics Committee and conformed to UK Home Office regulations under the Animals (Scientific Procedures) Act 1986 including Amendment Regulations 2012. Human biological samples were collected, stored, and managed by the Cordoba node belonging to the Biobank of the Andalusian Health Service (Servicio Andaluz de Salud — SAS) and approved by the Ethics and Clinical Research Committee of the University Hospital Reina Sofia. All subjects gave informed consent.

## Author contributions

Conceptualization was contributed by EJR, LL, MED, and AB. Methodology was contributed by JD. Formal analysis was contributed by GK. Investigation was contributed by EJR, LL, MED, AC, EMR, CDC, JDH, BSD, and EN. Resources were contributed by JDH and JPD. Writing and draft preparation were contributed by EJR, MED, and AB. Supervision was contributed by AB. Funding acquisition was contributed by AB.

## Supplementary Material

Supplemental data

## Version 1 07/08/2021

Electronic publication
