# Supplementary material for: JunD, not c-Jun, is the AP-1 transcription factor required for Ras-induced lung cancer
Source: JCI Insight. 2021 Jul 8;6(13):e124985. doi: 10.1172/jci.insight.124985 (PMC8410048; doi:10.1172/jci.insight.124985)
Supplement: Supplemental data [file jciinsight-6-124985-s015.pdf]

JunD, not c-Jun, is the AP-1 transcription factor required for Ras-induced lung cancer

E. Josue Ruiz[1], Linxiang Lan[1], Markus Diefenbacher[1,2], Eva Madi Riising[1,3], Clive Da Costa[1], Atanu Chakraborty[1,4], Joerg Hoeck[1,5], Bradley Spencer-Dene[6,7], Gavin Kelly[8], Jean-Pierre David[9], Emma Nye[6], Julian Downward[10], and Axel Behrens[1,11,12,13*]

[1]Adult Stem Cell Laboratory, The Francis Crick Institute, 1 Midland Road, London NW1 1AT, U.K.

[2]Present address: Theodor Boveri Institute, Biocenter, University of Würzburg, Am Hubland, 97074 Würzburg, Germany and Comprehensive Cancer Center Mainfranken, University of Würzburg, 97080 Würzburg, Germany.

[3]Present address: Samplix ApS, Mileparken 28, 2730 Herlev, Denmark.

[4]Present address: AstraZeneca, B310 Cambridge Science Park, Milton Road, Cambridge CB4 0WG, U.K.

[5]Present address: Boehringer Ingelheim, 900 Ridgebury Road, Ridgefield, CT 06877, U.S.A.

[6]Experimental Histopathology, The Francis Crick Institute, 1 Midland Road, London NW1 1AT, U.K.

[7]Present address: GlaxoSmithKline Non-Clinical Histology, Bioimaging, Stevenage, U.K.

[8]Bioinformatics and Biostatistics, The Francis Crick Institute, 1 Midland Road, London NW1 1AT, U.K.

[9]Institute of Osteology and Biomechanics, University Medical Center, Hamburg-Eppendorf, 20246 Hamburg, Germany.

[10]Oncogene Biology Laboratory, The Francis Crick Institute, 1 Midland Road, London NW1 1AT, U.K.

[11]Cancer Stem Cell Laboratory, Institute of Cancer Research, London, U.K.

[12]Imperial College, Division of Cancer, Department of Surgery and Cancer, U.K.

[13]Convergence Science Centre, Imperial College, London, SW7 2BU, U.K.

[*]Corresponding author: Axel Behrens, Cancer Stem Cell Laboratory, Institute of Cancer Research, 237 Fulham Road, London SW3 6JB, U.K. Phone: +44 (0) 20 71535250. Email: axel.behrens@icr.ac.uk

Short title: JunD is required for Ras-induced lung cancer

Supplemental Figure Legends

Supplemental Figure 1 (relates to Figure 1): Classification of lung tumors observed in lsl-KRasG12D and c-Junf/f; K-RasG12D mice.

(A) Quantification of lung tumor types in control (*K-Ras*^{G12D}, 4 mice) and c-Jun-deleted (*c-Jun*$^{\Delta/\Delta}$*; K-Ras*^{G12D}, 3 mice) mice at 12 weeks post-intubation. LADC, lung adenocarcinoma; LSCC, lung squamous cell carcinoma; SCLC, small cell lung cancer.

(B) Ki67 antibody stains of lung tumor sections from mice of the indicated genotypes, 12 weeks post-intubation. Arrowheads indicate Ki67-positive cells. Scale bars: 100µm.

(C) H&E stained sections of lung tumors (left panel) and quantification (right panel) of lung tumor grades identified in *K-Ras*^{G12D} and *c-Jun*$^{\Delta/\Delta}$*; K-Ras*^{G12D} mice. Scale bars: 50µm.

(D) H&E, TTF-1 and HMGA2 immunohistochemistry in *c-Jun*$^{\Delta/\Delta}$*; K-Ras*^{G12D} tumors. Scale bars: 100 µm.

(E) Example of lepidic predominant adenocarcinoma (formerly bronchioloalveolar carcinoma, BAC) lesions (enlarged in a' and b') found in *c-Jun*$^{\Delta/\Delta}$*; K-Ras*^{G12D} mice. Scale bars: 1 mm (left panel), 100 µm (right panels).

Supplemental Figure 2 (relates to Figure 2): Validation of tumorigenic potential of cultured *KP* and *JKP* tumor cells by orthotopic transplant into host mice.

(A) Ki67 antibody stains of lung tumor sections from *K-Ras*^{G12D}*; p53*$^{f/f}$ (*KP*) and *c-Jun*$^{f/f}$*; K-Ras*^{G12D}*; p53*$^{f/f}$ (*JKP*) mice, 10 weeks post-intubation. Scale bars: 200 µm.

(B) Experimental strategy: Eight-week-old Nu/Nu mice were administered with *KP* or *JKP* lung tumor cells by intratracheal intubation (i.t.) and lungs were analysed after 12 weeks.

(C) Quantification of tumor incidence in mice (n = 10 per group) receiving *KP* or *JKP* cells as in (B). Two independent cell clones were tested per genotype.

(D) H&E stains of lung sections from *KP* and *JKP* cell-receiving mice. Scale bars: 2 mm (whole sections), 50 µm (magnified areas).

(E) c-Jun and JunD antibody stains of lung tumors from *KP* and *JKP* cells. Scale bars: 5 µm.

(F) In vivo tumor graft growth curves of *KP* and *JKP* lung tumor cells subcutaneously injected in Nu/Nu mice (n = 5 each cell type). Graph shows means ± S.E.M.. P values calculated using Two-way ANOVA with Bonferroni's multiple comparisons test.

(G) Kaplan-Meier curves of *KP* and *JKP* lung tumor cells subcutaneously injected in Nu/Nu mice (n = 5 each cell type). Median survival: *JKP* 23 days, *KP* 36 days. P value calculated using log-rank (Mantel–Cox) test.

(H) Quantifications of immunoblot experiments display in figure 2I. Cultured *KP* and *JKP* cells were treated with or without anisomycin and JNK inhibitor (SP600125). The results are expressed as mean ± S.E.M.. P values calculated using Two-way ANOVA with Tukey's multiple comparisons test: *p<0.05; **p<0.01; ***p<0.001; ****p<0.0001.

Supplemental Figure 3 (relates to Figure 4): Scheme depicting the generation of Jnkk2-Jnk1 transgenic mice.

(A) Schematic of the JNKK2-JNK1 fusion construct, consisting of a 3xHA tag, the human JNKK2 cDNA, 5 Gly-Gly repeats and the human JNK1 cDNA. The JNKK2-

JNK1 fusion construct was inserted into the multiple cloning site of the pENTR1ATM-vector (Life Technologies).

(B) Targeting of the genomic ROSA26 locus with the JNKK2-JNK1 vector. In the pEntry clone, the JNKK2-JNK1 construct is flanked by lambda phage integrase recognition sites (attL) and thus can be efficiently inserted into the targeting vector carrying the corresponding heterotypic sites (attR). The targeting construct consists of a 5'-ROSA26 homology arm, a splice acceptor (SA) site, a PGK-neo-STOP cassette flanked by loxP sites (LSL), the JNKK2-JNK1 fusion construct, an IRES-eGFP reporter gene, a 3'-ROSA26 homology arm and a PGK-DTA selection cassette. Screening PCR was performed using the forward primer indicated by arrow 1, 5' of the targeting construct and the reverse primer indicated by arrow 2 in the 5' region of the targeting construct. After Cre-mediated recombination, the LSL-cassette is excised and the JNKK2-JNK1 fusion construct is expressed in the genomic ROSA26 locus. For genotyping PCR, primers indicated by arrows 3 and 4 located in the eGFP reporter gene were used. Arrowheads indicate loxP sites.

(C, D) Validation of the JNKK2-JNK1 transgene. Western blot analysis of protein extracts from control lsl-Jnkk2-Jnk1 heterozygous mice, or lsl-Jnkk2-Jnk1 heterozygous mice crossed to a Nestin-Cre line, showing the HA-tagged JNKK2-JNK1 fusion protein (97kDa) (C) and c-Jun phosphorylated at serine 73 (D).

(E) Ki67 antibody stains of lung tumor sections from mice of the indicated genotypes, 12 weeks post-i.t.. Scale bars: 200 µm.

(F) Phospho-JNKThr183/Tyr185 antibody stains of lung tumors from mice of the indicated genotypes. Scale bars: 50 µm.

Supplemental Figure 4 (relates to Figure 5): JunD is required for Ras-driven tumorigenesis.

(A) Kaplan-Meier plot showing the association between *JUND* expression and patient survival. Analysis performed using KM plotter lung cancer database.

(B) Ki67 antibody stains of lung tumor sections from mice of the indicated genotypes, 12 weeks post-intubation. Arrowheads indicate Ki67-positive cells. Scale bars: 200µm.

(C) Cell proliferation was measured in cultured *KP* and *KPD* cells. Graph shows means ± s.d.. ***P<0.001, ****P<0.0001, P values calculated using Two-way ANOVA with Bonferroni's multiple comparisons test.

(D, E) In vivo tumor graft growth curves of *KP* and *KPD* cell lines subcutaneously injected in opposite flanks of immunocompromised mice (n = 5). Graph shows means ± S.E.M.. ****P = $1.8x10^{-14}$, P values calculated using Two-way ANOVA with Bonferroni's multiple comparisons test (D). Plots showing the weight of xenograft tumors at the end point. Mean is shown; P values calculated using Unpaired t-test with Welch's correction (E).

(F) Immunoblot analysis and quantification (G) of cultured *KP* and *KPD* cells probed for phospho-c-JunSer63, phospho-c-JunSer73/phospho-JunDSer100, c-Jun, JunD, phospho-JNKThr183/Tyr185 and JNK. Tubulin is shown as loading control. The results are expressed as mean ± S.E.M. (n = 3 per group). P values calculated using Unpaired t-test with Welch's correction.

Supplemental Figure 5 (relates to Figure 5): siRNA-mediated knockdown of JUND reduced cell growth through apoptotic cell death in human LADC cell lines

(A) Graphs showing the difference in cell proliferation between control (Scramble) and siJUND-transfected human LADC cell lines (NCI-H23, NCI-H1792, NCI-H441 and

A549). Graphs indicate mean ± S.E.M.. P values calculated using one-way ANOVA with Tukey's multiple comparisons test.

(B) siRNA-mediated knockdown of JUND decreases c-Jun protein and phospho-c-Jun levels in human LADC cell lines. Vinculin is shown as loading control.

(C) Impaired cell growth in siJUND-transfected LADC cell lines is associated with apoptotic cell death based on the DEVD-NucView488 Caspase-3 Dye that detects caspase-3 activity. Scale bars: 400 µm.

Supplemental Figure 6 (relates to Figure 5): p38α MAPK is active in c-Jun deficient cells.

(A) Gene set enrichment analysis of transcription data from *KP* and *JKP* cells.

(B) Immunoblot analysis of cultured *KP* and *JKP* cells probed for phospho-p38α, p38α and c-Jun. Tubulin is shown as loading control.

(C) Immunoblot analysis of cultured *KP, KDP* and *JKP* cells probed for phospho-p38α, p38α, c-Jun and JunD. Tubulin is shown as loading control.

(D) *KP* and *JKP* cells were cultured with or without p38α inhibitor (SB203580), and proliferation was measured. Graph shows means ± s.d.; ****$P<0.0001$, P values calculated using Two-way ANOVA with Tukey's multiple comparisons test.

(E) Positive correlation of mRNA expression of *JUND* and *MAPK14* in human lung adenocarcinoma. R: Spearman's correlation coefficient. Transcripts per million (TPM).

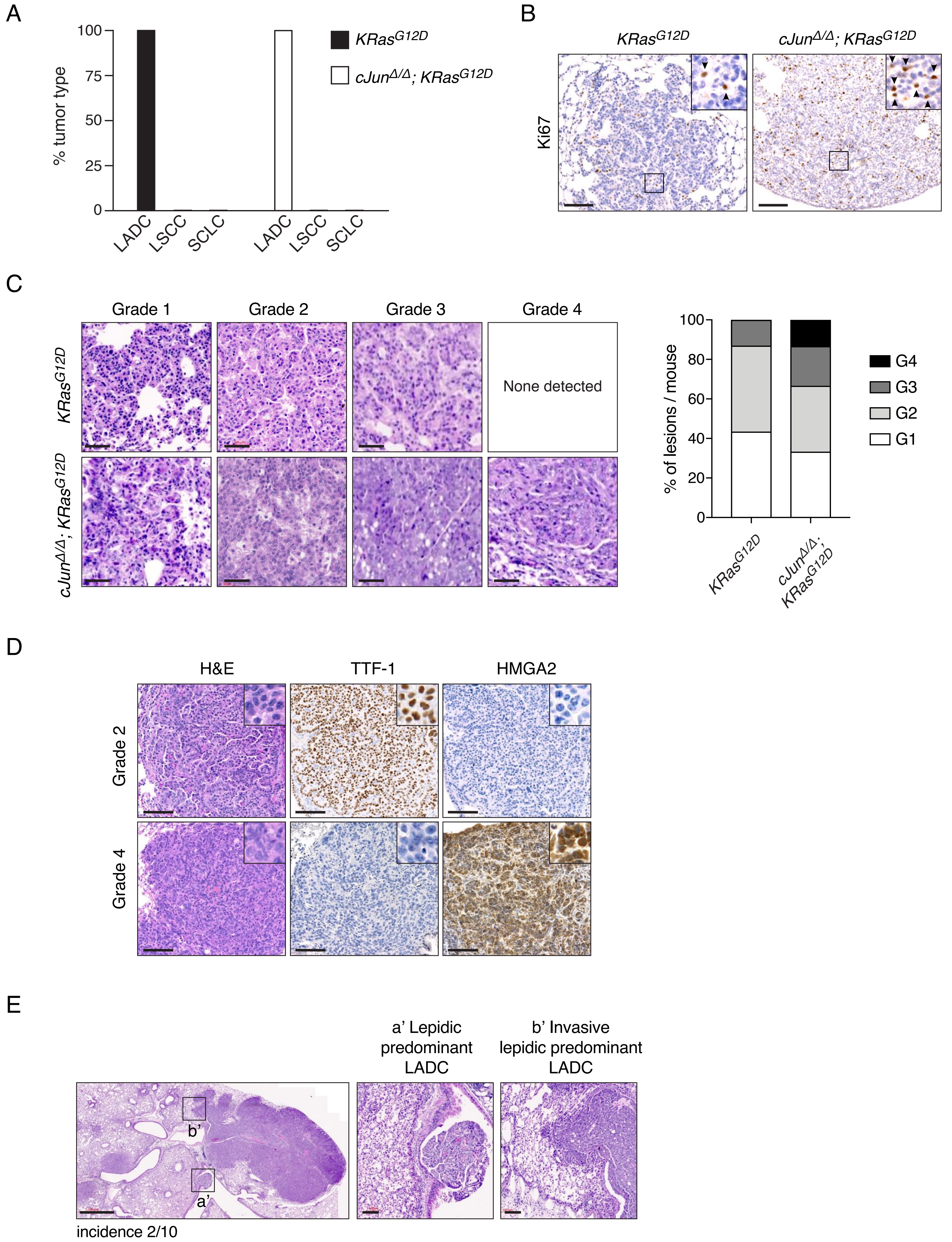

Supplemental Figure 1

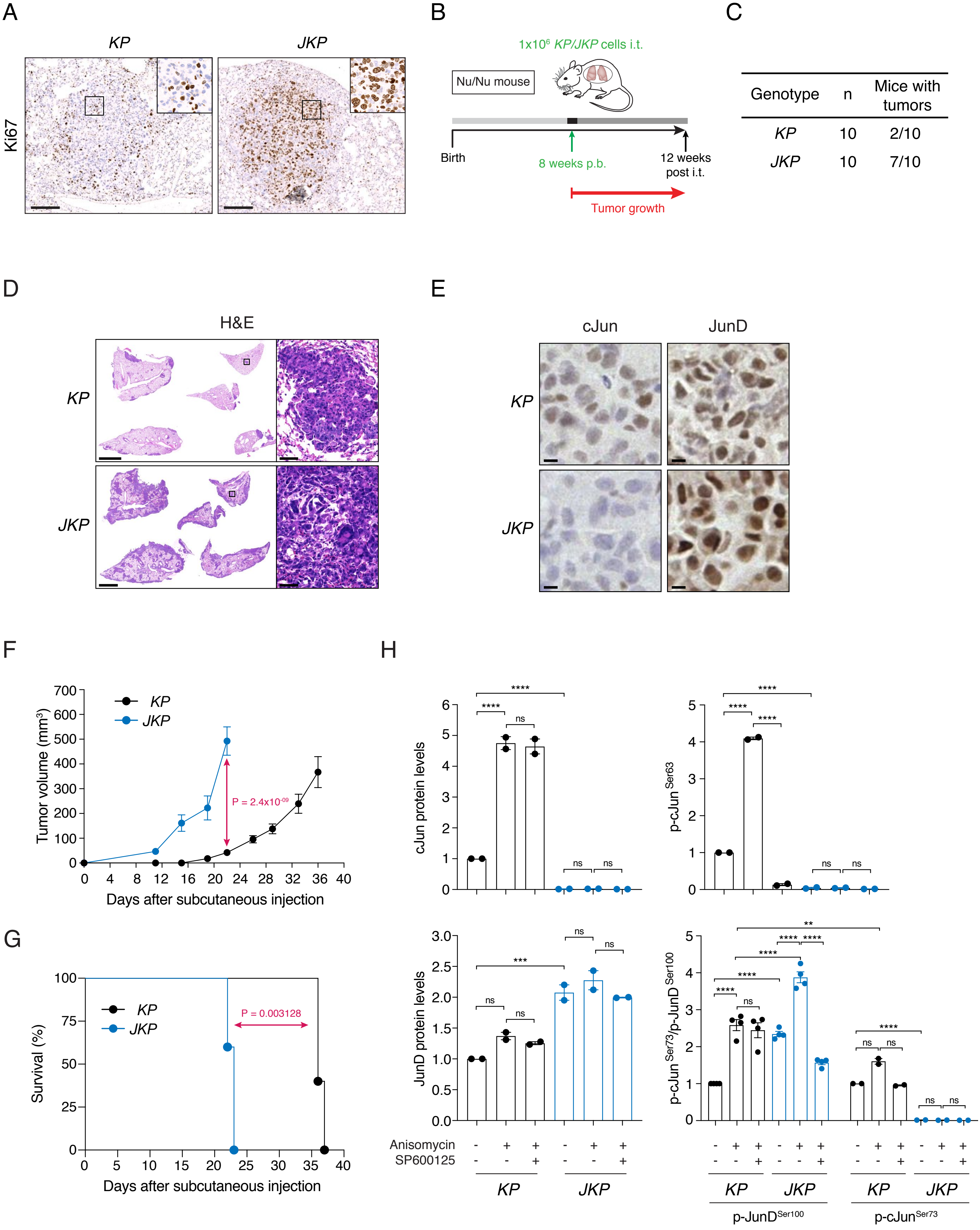

Supplemental Figure 2

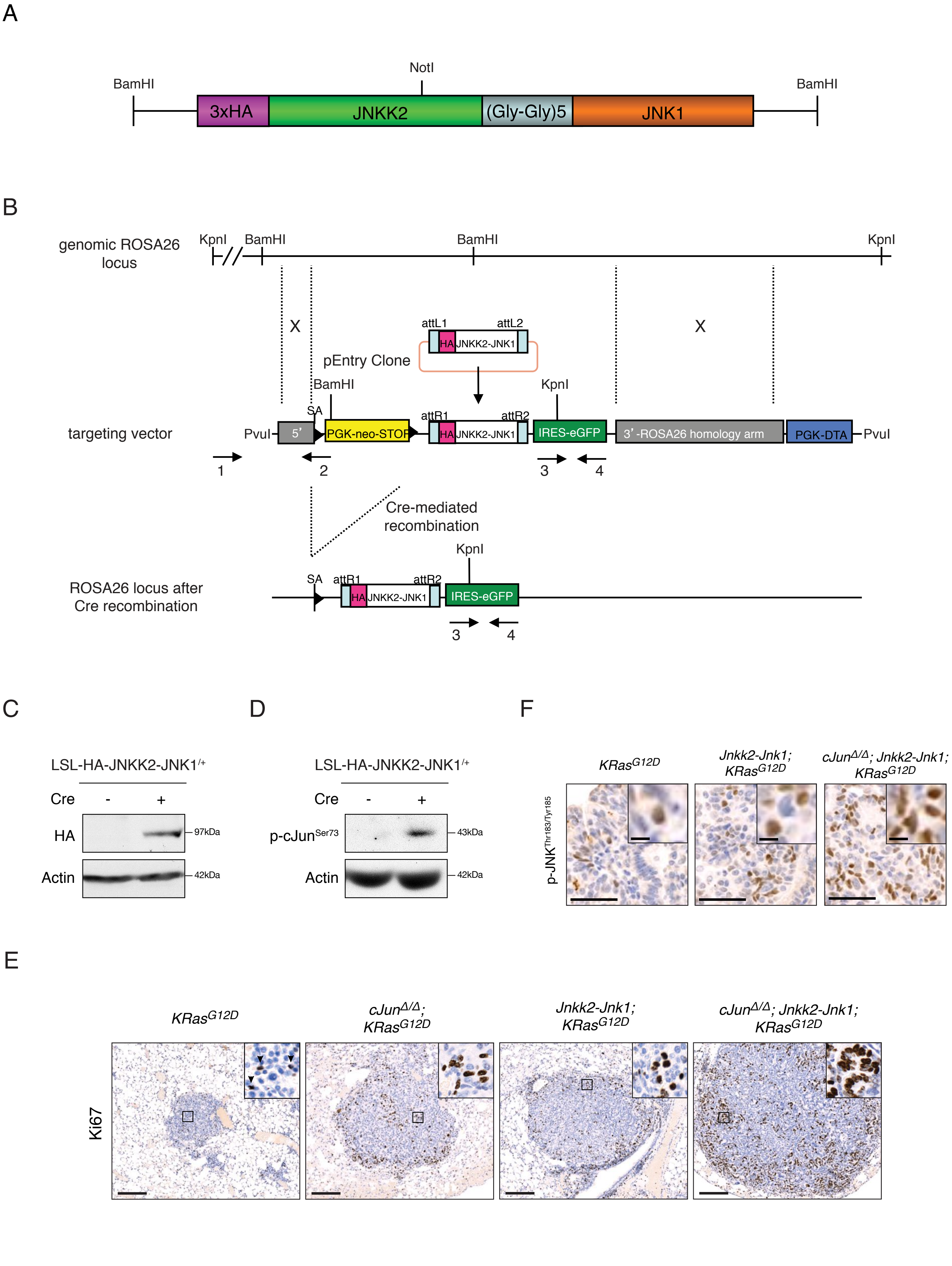

Supplemental Figure 3

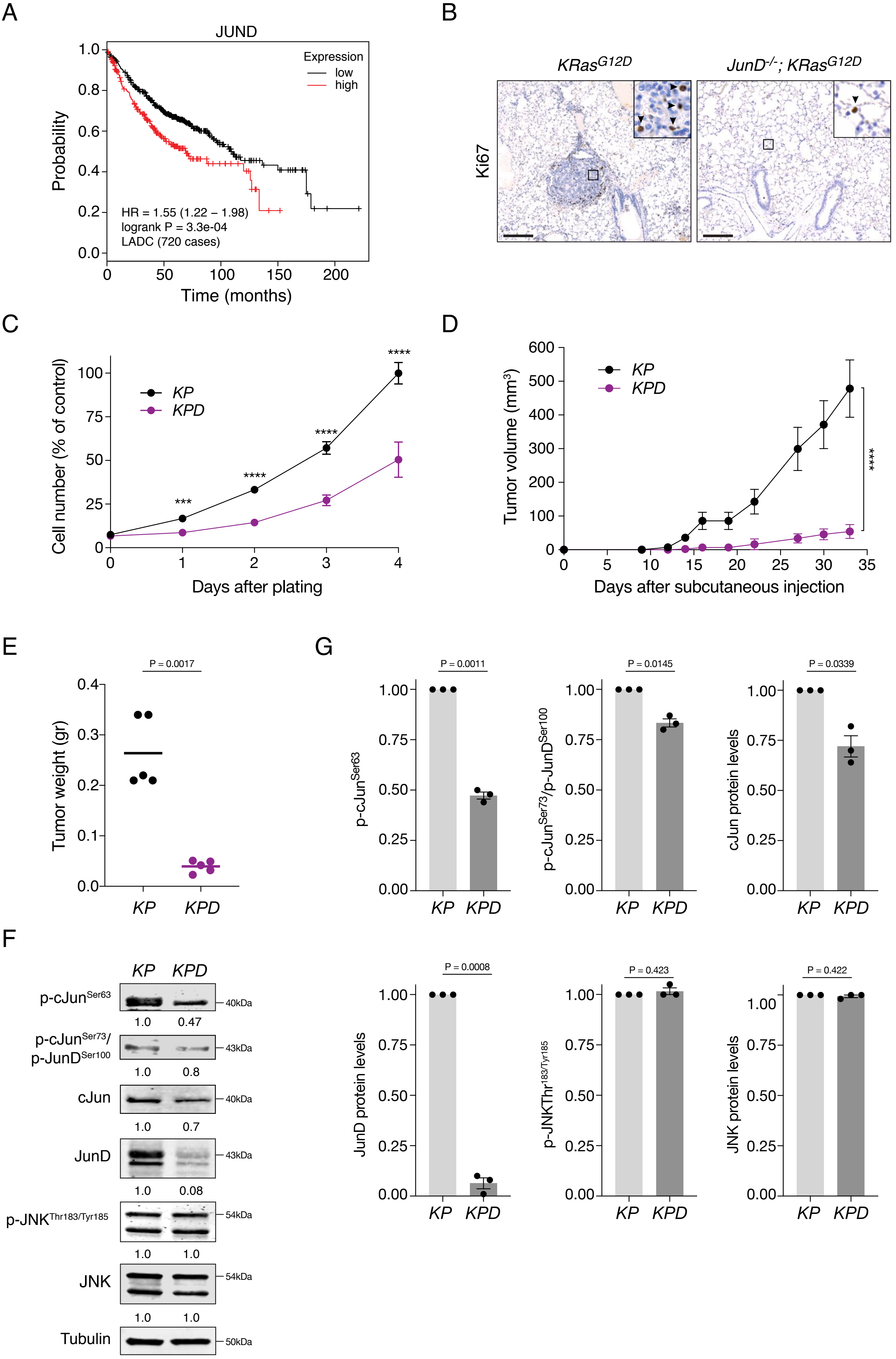

Supplemental Figure 4

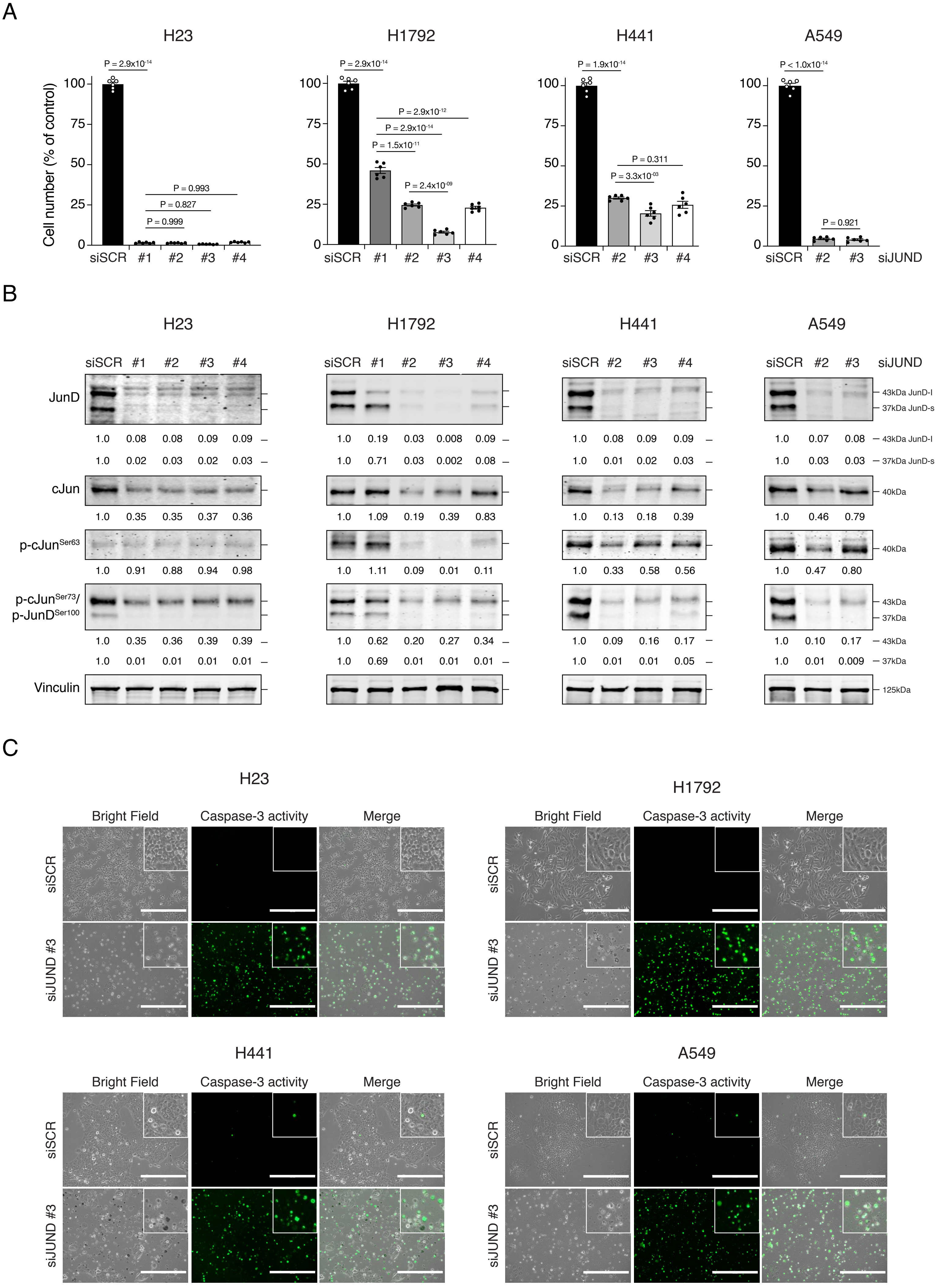

Supplemental Figure 5

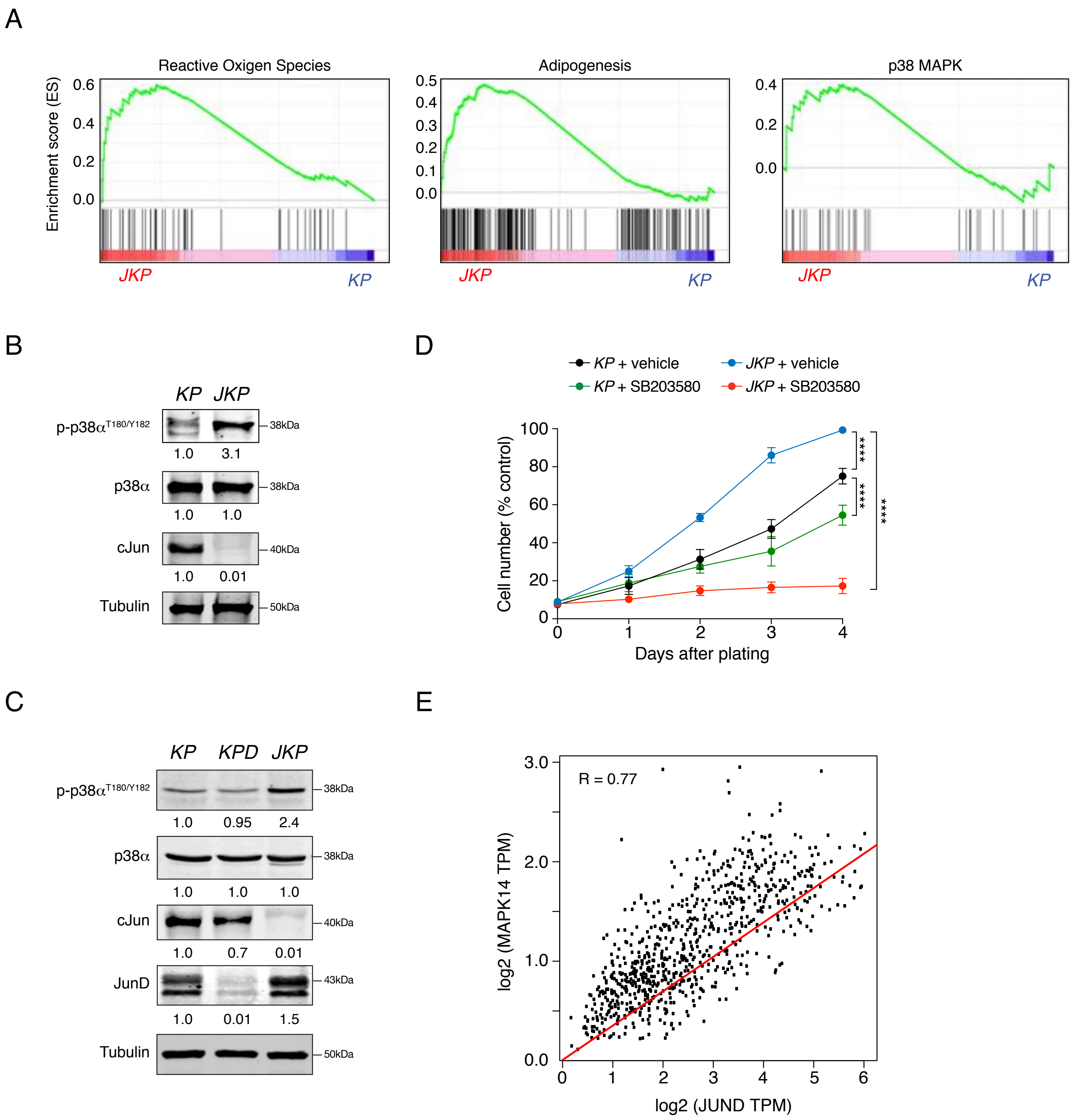

Supplemental Figure 6
